# Efficacy and Safety of “Vacuum Swallowing” Based on a Strong Negative Esophageal Pressure in Healthy Individuals

**DOI:** 10.1007/s00455-024-10741-y

**Published:** 2024-08-17

**Authors:** Kenjiro Kunieda, Saori Suzuki, Satoe Naganuma, Keishi Okamoto, Tomohisa Ohno, Takashi Shigematsu, Naomi Yagi, Yoshitaka Oku, Ichiro Fujishima

**Affiliations:** 1 Department of Neurology, Gifu University Graduate School of Medicine, 1-1 Yanagido, Gifu, Gifu 501-1194 Japan; 2 Department of Rehabilitation Medicine, Hamamatsu City Rehabilitation Hospital, Hamamatsu, Shizuoka Japan; 3 Department of Rehabilitation, Hamamatsu City Rehabilitation Hospital, Hamamatsu, Shizuoka Japan; 4 Department of Dentistry, Hamamatsu City Rehabilitation Hospital, Hamamatsu, Shizuoka Japan; 5 Advanced Medical Engineering Research Institute, University of Hyogo, Himeji, Hyogo Japan; 6 Department of Physiology, Hyogo Medical University, Nishinomiya, Hyogo Japan

**Keywords:** Deglutition disorders, Esophagus, Inspiratory muscles, Lateral medullary syndrome, Lower esophageal sphincter, Manometry

## Abstract

Vacuum swallowing is a unique method for improving the pharyngeal passage of a bolus by creating subatmospheric negative pressure in the esophagus. However, whether healthy individuals and other patients with dysphagia can reproduce vacuum swallowing remains unclear. Therefore, this study aimed to assess whether healthy individuals verified using high-resolution manometry (HRM) could reproduce vacuum swallowing and evaluate its safety using a swallowing and breathing monitoring system (SBMS). Two healthy individuals who mastered vacuum swallowing taught this method to 12 healthy individuals, who performed normal and vacuum swallowing with 5 mL of water five times each. The minimum esophageal pressure and the maximum pressure of the lower esophageal sphincter (LES) were evaluated during each swallow using the HRM. Additionally, respiratory-swallowing coordination was evaluated using the SBMS. Ten individuals reproduced vacuum swallowing, and a total of 50 vacuum swallows were analyzed. The minimum esophageal pressure (-15.0 ± 4.9 vs. -46.6 ± 16.7 mmHg; *P* < 0.001) was significantly lower, and the maximum pressure of the LES (25.4 ± 37.7 vs. 159.5 ± 83.6 mmHg; *P* < 0.001) was significantly higher during vacuum swallowing. The frequencies of the I-SW and SW-I patterns in vacuum swallowing were 38.9% and 0%, respectively, using the SBMS. Vacuum swallowing could be reproduced safely in healthy participants with instruction. Therefore, instructing exhalation before and after vacuum swallowing is recommended to prevent aspiration.

**Supplementary Information:**

The online version contains supplementary material available at 10.1007/s00455-024-10741-y.

## Introduction

Dysphagia is one of the most prevalent and distressing symptoms that results in severe complications, such as aspiration pneumonia, choking, malnutrition, dehydration, and poor quality of life [1, 2]. Various swallowing methods have been developed for patients with dysphagia, and balloon dilatation, Shaker exercise, botulinum toxin type A injection, and cricopharyngeal myotomy are reportedly beneficial for improving the passage of bolus through the upper esophageal sphincter (UES) relaxation [3–8]. However, posture adjustments, such as reclining, chin tuck, and head rotation, are frequently used as compensatory swallowing techniques to lower the risk of aspiration and pharyngeal residual materials [9–14].

A previous study reported a case of a patient with lateral medullary syndrome (Wallenberg’s syndrome) and severe bulbar dysphagia who acquired a unique swallowing method to improve the pharyngeal passage of a bolus by creating a strong subatmospheric negative pressure in the esophagus [15]. This method was named “vacuum swallowing.” Furthermore, the authors reported that some patients spontaneously acquired vacuum swallowing in the recovery process of bulbar-type dysphagia due to lateral medullary syndrome (LMS). Vacuum swallowing may be a compensatory method observed in individuals recovering from LMS-induced dysphagia [15, 16]. This swallowing method was reported as a novel alternative swallowing maneuver for dysphagia caused by LMS [15–19]. The concept of vacuum swallowing is novel in creating a pressure gradient between the weak pharyngeal and intra-esophageal negative pressures. Bulbar-type dysphagia leads to residue in the pharynx, particularly the pyriform sinus, because of the weak pharyngeal contraction and impaired UES relaxation. Therefore, vacuum swallowing might be effective in removing hypopharyngeal residues.

Furthermore, the mechanism of this swallowing method was assessed using high-resolution manometry (HRM) [15, 16, 19, 20], which revealed a strong subatmospheric esophageal pressure generation with constriction of the lower esophageal sphincter (LES) muscles. Expansion of the intercostal space and contraction of the diaphragm during vacuum swallowing was observed on videofluoroscopic examination of swallowing (VF) [15]. During vacuum swallowing, the contraction of the inspiratory muscles, including the diaphragm and external intercostal muscles, might be involved in creating a strong subatmospheric negative pressure in the thoracic cavity. The mechanism underlying the pharyngeal passage of bolus improvement was the pressure gradient created between the pharynx and esophageal body using the inspiratory muscles during swallowing. Reports on swallowing pressure changes during recovery from dysphagia due to brainstem stroke exist [21]. However, several reports on subatmospheric negative pressure creation in the esophagus during stroke recovery are also available.

In acquiring vacuum swallowing, the risk of aspiration of bolus should be evaluated because of the inspiratory effort made during swallowing. Aspiration of hypopharyngeal residues due to dysphagia, such as the bulbar type, should also be prevented before and after vacuum swallowing. Furthermore, confirming that airflow into the lower airway does not occur during vacuum swallowing is necessary. Therefore, the breathing pattern before and after swallowing is essential to prevent adverse events, such as aspiration [21–23]. Notably, a non-invasive and quantitative technique for evaluating breathing-swallowing coordination was developed [21, 23]. Breathing and swallowing can be monitored as a time series, and the respiratory phase sequence, overlap, and delay of inspiration/expiration and swallowing can be identified. Moreover, the risk of aspiration related to vacuum swallowing has been assessed with a swallowing and breathing monitoring system (SBMS), which can be used to evaluate the aspiration risk based on the coordination of swallowing and breathing [22, 24, 25].

However, whether healthy individuals and other patients with dysphagia can reproduce vacuum swallowing remains unclear. It might be useful as a new swallowing method for other dysphagia patients. We hypothesized that healthy individuals could safely reproduce vacuum swallowing with instruction. Therefore, this study aimed to assess whether healthy individuals being verified using the HRM can reproduce vacuum swallowing and evaluate the safety of vacuum swallowing by assessing the coordination of breathing and vacuum swallowing with an SBMS.

## Methods

### Participants

In total, 12 healthy individuals were recruited for participation in this study (mean age, 30.5 ± 5.9 years; seven males). The study protocol was explained to all participants, who gave verbal and written consent before enrollment. None of the participants had a history of dysphagia, gastrointestinal disease, pulmonary and neurological diseases, or other significant medical conditions. The experimental protocol was approved by the Ethics Committee of *Hamamatsu City Rehabilitation Hospital* (#17 − 02).

#### High-Resolution Manometry (HRM)

HRM was used to determine whether the participants successfully reproduced vacuum swallowing. Pressure and timing data were extracted using the ManoScan software (Medtronic Inc., Minneapolis, MN, USA), and a solid-state high-resolution manometer (Laborie Medical Technologies Corp., Portsmouth, NH, USA) was used for data collection. Manometric data were obtained using a solid-state manometry catheter assembly (outer diameter, 4.2 mm) with 36 circumferential pressure sensors placed 1 cm apart. The catheter was calibrated and zeroed to atmospheric pressure, and manometric studies were performed in the sitting position. The nasal cavity was anesthetized with lidocaine jelly. Additionally, the catheter was lubricated with lidocaine jelly to ease its passage through the nasal cavity; it was intranasally inserted and positioned to obtain data from the hypopharynx to the stomach beyond the high-pressure zone at the LES. Next, the catheter was fixed with tape at the nasal wing. After a 5–10-min acclimatization period, the basal pressure was recorded for 30 s. Subsequently, the participants reproduced the vacuum swallowing technique as per the instructions.

### Instruction on Vacuum Swallowing

First, two healthy individuals, the attending medical doctor and speech-language pathologist for the previously reported case, attempted to reproduce vacuum swallowing by observing the patient and VF [15]. The VF revealed diaphragmatic movement and thoracic expansion during vacuum swallowing. As previously reported, these two instructors confirmed that the use of HRM created strong subatmospheric negative pressure in the esophagus during swallowing. Consistent with previous reports, strong negative pressure creation in the esophagus and increased LES pressure were observed during vacuum swallowing compared to normal swallowing.

**Fig. 1:**
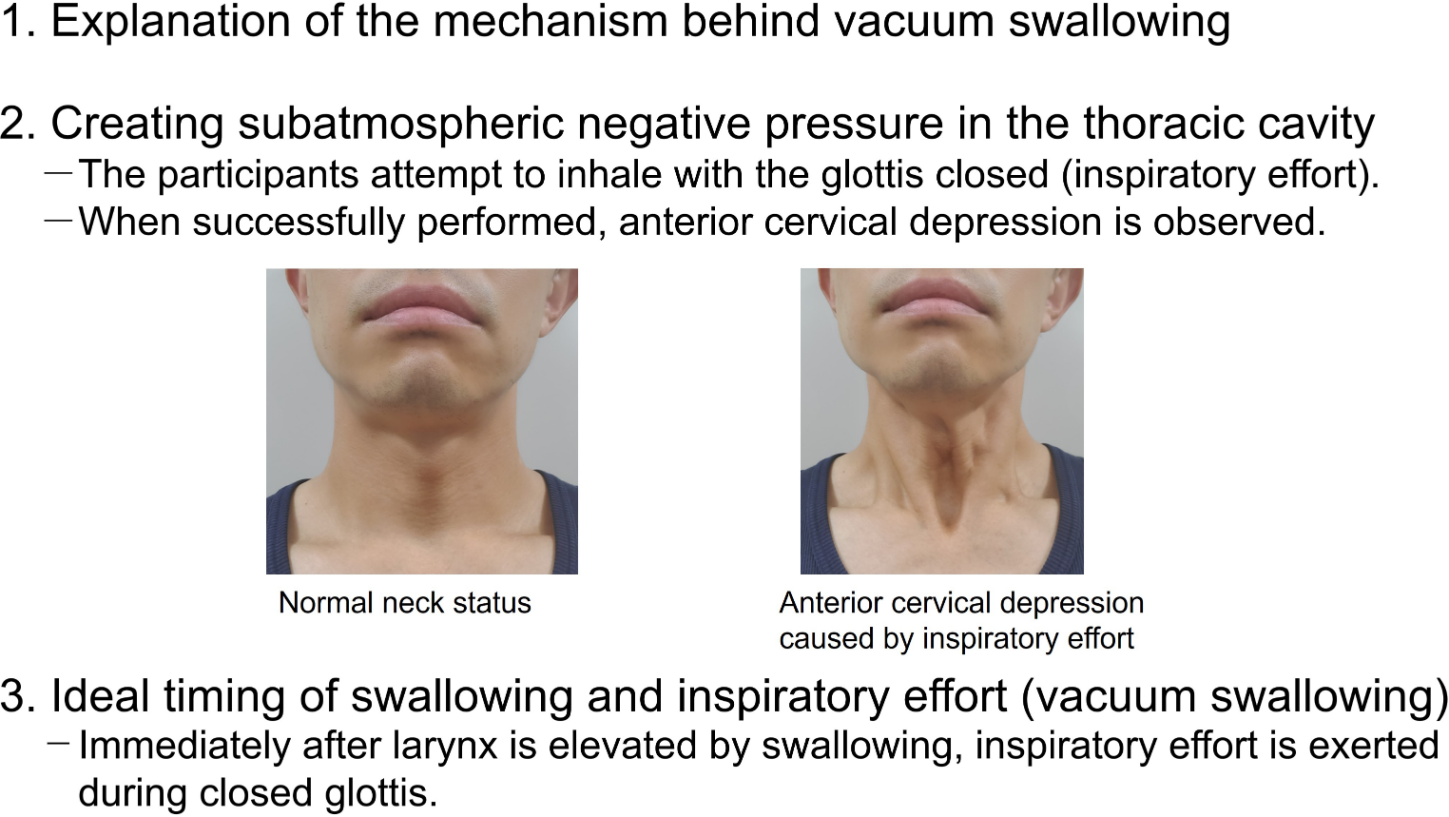
Procedure for the instruction of vacuum swallowing. After explaining the mechanism behind vacuum swallowing, two points need to be instructed: First, how to create subatmospheric negative pressure in the thoracic cavity; second, the ideal timing of swallowing and inspiratory effort. The depression of the neck indicates the generation of strong negative pressure in the thoracic cavity

The two instructors who acquired vacuum swallowing taught the swallowing technique to the remaining 12 participants. First, they explained the mechanism of the swallowing method for approximately 20 min using a video portraying vacuum swallowing. The video included the VF findings and HRM’s topography in vacuum swallowing. Table 1; Fig. 1 present the instructions on vacuum swallowing. Furthermore, a video for instruction is provided in Online Resource 1.

**Table 1:** Instructions for vacuum swallowing (referring to Online Resource 1)

Explanation of the mechanism behind vacuum swallowing	The participants need to understand three points: 1, how to create subatmospheric negative pressure in the thoracic cavity; 2, the ideal timing of swallowing and inspiratory effort; and 3, the prevention of aspiration during vacuum swallowing. A video of vacuum swallowing can be used to help understand vacuum swallowing^a^. During swallowing, the glottis is closed. Furthermore, the mouth is closed, the tongue is in contact with the palate, and the pharynx is contracted. Therefore, air does not flow into the airway due to inspiratory effort during swallowing, and intrathoracic subatmospheric negative pressure is created.
Creating subatmospheric negative pressure in the thoracic cavity	The participants attempt to inhale with the glottis closed (inspiratory effort). Inspiratory efforts with the opened mouth would be useful during vocal cord closure to create subatmospheric negative pressure in the thoracic cavity. Alternatively, participants attempt to inhale with their mouths closed. These techniques may be repeated. When successfully performed, a strong subatmospheric negative pressure in the thoracic cavity is created due to the contraction of the inspiratory muscles, and anterior cervical depression is observed. These cervical findings can be checked with a mirror by the participants. Imaging or demonstrating the inability to inhale due to choking may be one of the easier teaching methods to understand the creation of intrathoracic strong subatmospheric pressure. If the creation of intrathoracic subatmospheric negative pressure is difficult with the above process, they might attempt an inhalation effort by pinching their nose.
Ideal timing of swallowing and inspiratory effort	The participants exhale and hold their breath before vacuum swallowing. Immediately after the larynx is elevated (glottis closed) by swallowing, inspiratory effort, as described above, is exerted during closed glottis. Visual feedback with a mirror to confirm these findings is useful. Combining prolonged laryngeal elevation as Mendelsohn maneuver and inspiratory effort may help the patient understand the ideal timing of swallowing and inspiratory effort. With the mouth closed and the tongue in contact with the palate immediately after swallowing, no airflow into the airway occurs. Neck depression was assessed to confirm whether the swallowing technique was successfully reproduced. Visual feedback with a mirror to confirm these findings is also useful.
Instructions to prevent aspiration	The pattern of coordination between breathing and vacuum swallowing is the expiration-vacuum swallowing-expiration pattern. Patients hold their breath before vacuum swallowing and exhale after vacuum swallowing. Supraglottic swallow might be useful for instruction.

^a^A video of vacuum swallowing is available in an open-access article [15]

### Swallowing Monitoring System and Analysis Methods

**Fig. 2:**
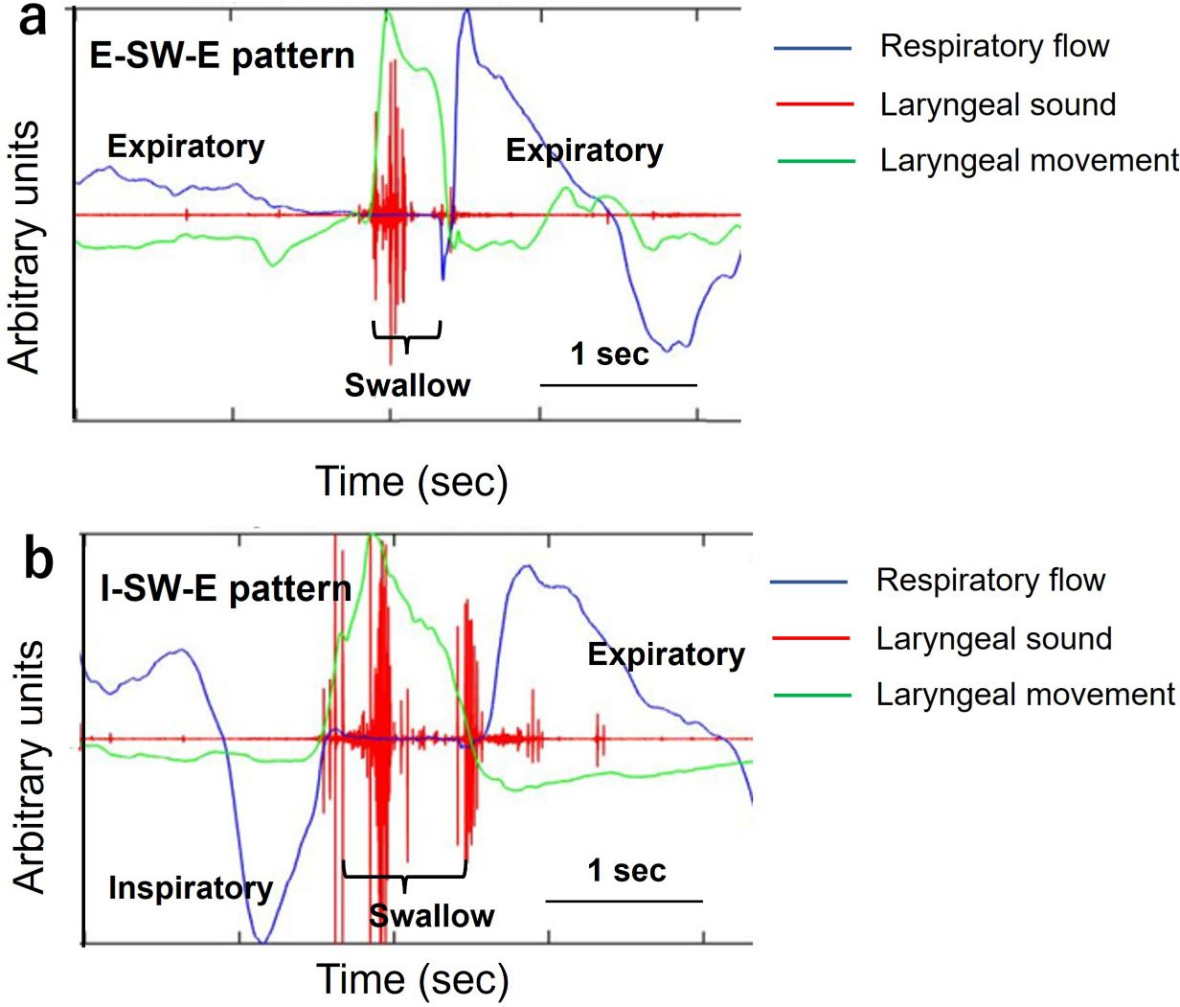
Coordination between breathing and swallowing. Swallowing occurs during expiration, and respiration resumes with expiration (a: E–SW–E pattern). Swallowing is immediately followed by inspiration, and expiration occurs after swallowing (b: I-SW-E pattern). The values on the y-axis were analog-digital converted values of biological signals obtained from respiration and laryngeal elevation E–SW–E, expiration–swallow–expiration; I-SW-E, inspiration-swallowing-expiration

We used an SBMS to evaluate its safety using respiratory flow, laryngeal motion, and swallowing sound [22, 24, 25]. The monitoring system comprises a nasal cannula-type flow sensor, a film-type piezoelectric sensor, and signal processing units. The laryngeal motion and the absence of respirator flow indicated swallows. Using a series of respiratory flow, the laryngeal motion, and sound data, the piezoelectric sensor has a wide dynamic range (0–4 kHz), which ensures the capture of both the laryngeal motion and sound. Swallowing periods are extracted semi-automatically with an algorithm using the respiratory flow, the swallowing sound, and the laryngeal motion [25]. We evaluated breathing–swallowing coordination and respiratory pause during swallowing. Figure 2 shows an example of a breathing–swallowing coordination analyzed by this system. Swallowing typically occurs during expiration, and the subsequent respiration re-initiates with expiration [23–25]. The expiration-swallow-expiration (E-SW and SW-E) pattern is usual. Vacuum swallowing creates a subatmospheric negative pressure in the thoracic cavity through inspiratory effort; therefore, the risk of aspiration might be high. The E-SW/SW-E patterns are useful to prevent the pharyngeal residues from invading the lower airway [24]. However, two unusual patterns (I-SW and SW-I) may also occur in healthy individuals [24, 25], and their occurrence (I-SW and/or SW-I) may indicate breathing-swallowing discoordination. The frequency of I-SW and SW-I patterns increases with age in patients with stroke, head-neck cancer after treatment, Parkinson’s disease, and chronic obstructive pulmonary disease [25]. Although these patterns are observed even in healthy individuals, the identification of a high I-SW/SW-I rate and the appropriate treatment to reduce it may prevent aspiration pneumonia [25]. We used water for test foods. The participants were upright, sitting on a chair, and voluntarily swallowed approximately 3 mL water from a 10-mL syringe three times during each normal and vacuum swallowing. These swallowing and respiratory patterns were identified using automatic calculations.

### Data Collection

**Fig. 3:**
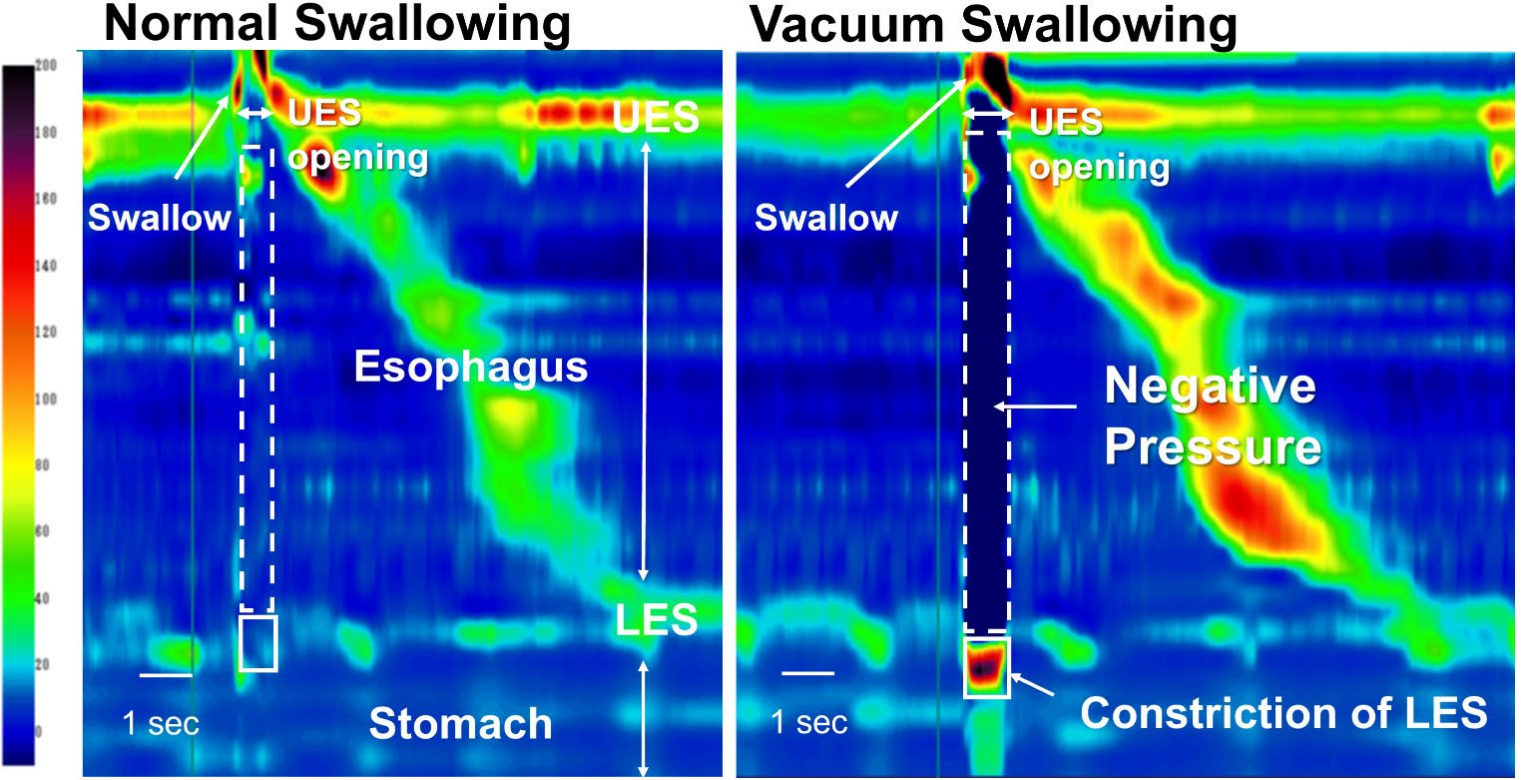
Pressure topography of healthy participants using high-resolution manometry. The subatmospheric pressure in the esophagus was defined as the minimum pressure between the UES and the LES when the UES opened due to swallowing. The maximum pressure of the LES was defined as the LES pressure during swallowing. Vacuum swallowing (right) is characterized by creating subatmospheric negative pressure in the esophageal body and constriction of LES. LES, lower esophageal sphincter; UES, upper esophageal sphincter

HRM data were analyzed using the ManoView analysis software (Star Medical, Tokyo, Japan). The minimum esophageal pressure (Pmin) and the maximum pressure (Pmax) of the LES were measured during swallowing (Fig. 3). First, the UES opening time during swallowing was used as the range of analysis on the time axis. Subsequently, the Pmin during swallowing was assessed within the analysis range defined between the upper and lower ends as the UES and LES, respectively. Regarding the Pmax during swallowing, it was analyzed at the resting LES level. During vacuum swallowing, Pmax was evaluated, including the high-pressure zone of the LES. The participants performed vacuum and normal swallowing with 5 mL of water five times each. Visual feedback was also provided using the HRM topography to enable the participants to match the timings of swallowing with that of the LES contraction. Successful vacuum swallowing was defined as when the HRM topography simultaneously generated a strong esophageal negative pressure with swallowing.

### Statistical Analysis

The distribution of all continuous variables was assessed using the Shapiro–Wilk test. Comparisons with and without vacuum swallowing were conducted using the paired t-test and Wilcoxon signed-rank test for normally and non-normally distributed continuous variables, respectively. The collected data were compared between normal and vacuum swallowing using paired *t*-tests. Furthermore, the critical value for rejecting the null hypothesis was *P* < 0.05. All statistical analyses were performed using IBM SPSS Statistics for Windows, version 22.0 (IBM Japan Corp., Tokyo, Japan).

## Results

Two of the 12 healthy participants could not tolerate catheter insertion and dropped out of the study.

### Esophageal Pressure Characteristics during Vacuum Swallowing

Ten healthy participants performed normal and vacuum swallowing five times each, and a total of 50 normal and vacuum swallows were analyzed. HRM revealed that the Pmin was significantly lower (*P* < 0.001), and the Pmax (*P* < 0.001) of the LES was significantly higher during vacuum swallowing than during normal swallowing (Table 2).

**Table 2:** Minimum esophageal pressure and the maximum pressure of the lower esophageal sphincter (LES) during vacuum and normal swallowing in healthy individuals

	Normal swallowing	Vacuum swallowing	Degrees of freedom	*P*-value	Effect size	Previously reported patient values [14]
Minimum esophageal pressure Pmin (mmHg)	−15.0 ± 4.9	−46.6 ± 16.7	9	< 0.001	0.92	−73.4
Maximum LES pressure Pmax (mmHg)	25.4 ± 9.8	152.7 ± 79.3	9	< 0.001	0.87	193.7

means ± standard deviation

### External Bodily Changes during Vacuum Swallowing

**Fig. 4:**
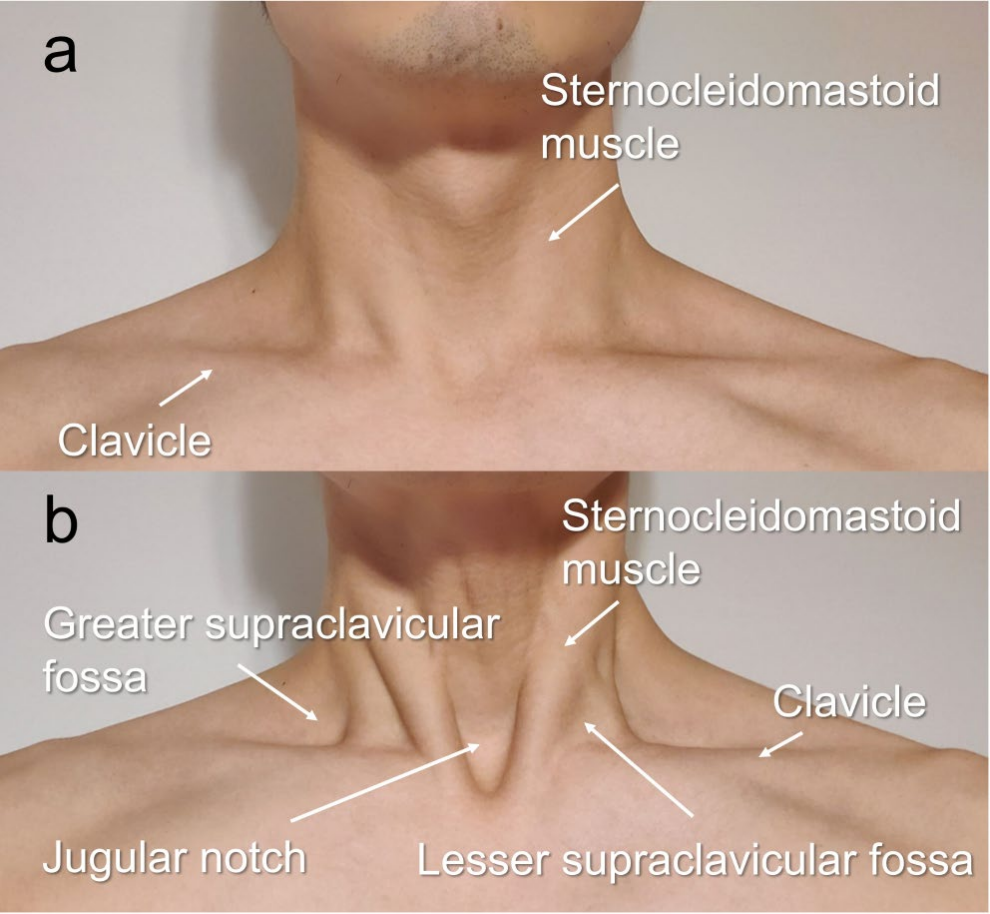
External bodily changes during vacuum swallowing　 **a** Normal neck status **b** Neck status during vacuum swallowing The sternocleidomastoid muscle and clavicle are relatively prominent. Neck depression reflects the strong subatmospheric negative pressure in the thoracic cavity

During vacuum swallowing, the sternocleidomastoid muscle and clavicle became prominent, reflecting the strong negative pressure in the thoracic cavity (Fig. 4). These findings were observed in all participants during vacuum swallowing. However, the depth of the cervical depressions detected by the instructor varied.

### Adverse Events

Two participants experienced mild coughing during one vacuum swallowing each. However, no other adverse events were recorded.

### Correlations Between Breathing-Swallowing Coordination

Six participants were assessed using an SBMS. The frequencies of the I-SW and SW-I patterns in vacuum swallowing were 38.9% and 0%, respectively. These patterns were 10.4% and 0% in normal swallowing, respectively (Tables 3 and 4). All participants showed respiratory pause during vacuum swallowing. However, no one choked on the water during vacuum swallowing in the SBMS study.

**Table 3:** Comparisons of frequencies of breathing-swallowing patterns between normal and vacuum swallowing

	Normal swallowing	Vacuum swallowing
E-SW-E	83.3%	61.1%
I-SW-E	16.7%	38.9%
E-SW-I	0%	0%
I-SW-I	0%	0%

E–SW–E, expiration–swallow–expiration; I-SW-E, inspiration-swallowing-expiration

**Table 4:** Breathing-swallowing patterns between normal and vacuum swallowing in six participants

	Normal swallowing	Vacuum swallowing
Participant 1	E-SW-E E-SW-E E-SW-E	E-SW-E E-SW-E E-SW-E
Participant 2	E-SW-E E-SW-E E-SW-E	I-SW-E E-SW-E E-SW-E
Participant 3	E-SW-E E-SW-E E-SW-E	E-SW-E I-SW-E E-SW-E
Participant 4	I-SW-E E-SW-E E-SW-E	E-SW-E E-SW-E E-SW-E
Participant 5	I-SW-E E-SW-E I-SW-E	E-SW-E I-SW-E I-SW-E
Participant 6	E-SW-E E-SW-E E-SW-E	I-SW-E I-SW-E I-SW-E

E–SW–E, expiration–swallow–expiration; I-SW-E, inspiration-swallowing-expiration

## Discussion

This is the first study to clarify that healthy individuals could reproduce the vacuum swallowing method verified by HRM analysis. The most important finding is that healthy participants could create a subatmospheric negative pressure in the esophagus during swallowing. Furthermore, the coordination of vacuum swallowing was assessed to ensure its safety and prevent respiratory complications, such as aspiration.

Interestingly, HRM showed constriction of the LES muscles and the synchronous generation of a strong subatmospheric negative pressure in the esophagus during vacuum swallowing. The Pmax of the LES was significantly higher during vacuum swallowing than during normal swallowing. Consequently, the increased LES pressure might reflect the contraction of the diaphragm, similar to that observed during deep inspiration [15]. The LES is composed of intrinsic (comprising esophageal muscle fibers and is under neurohormonal control) and extrinsic (consisting of the diaphragmatic crura and the phrenoesophageal ligament, which provide anatomical support to the LES) components [26]. Therefore, the diaphragm is the main component of the LES that contracts voluntarily during vacuum swallowing. In a previous study, the video of the VF in a patient showed diaphragmatic contraction and thoracic expansion during vacuum swallowing [15]. Therefore, the diaphragm and external intercostal muscles, which are necessary for inhalation, might contract during vacuum swallowing. Furthermore, when instructions for vacuum swallowing were provided, the participants were examined for a prominent sternocleidomastoid muscle and clavicle due to the subatmospheric negative pressure in the thoracic cavity. These findings suggest that healthy individuals employ contraction of the inspiratory and accessory muscles of respiration for successful vacuum swallowing.

Breathing patterns before and after vacuum swallowing are vital to protect the airway. Additionally, to prevent the aspiration risk of hypopharyngeal residues during vacuum swallowing, the expiration before and after swallowing is important. In cases of inhalation before or after swallowing, a risk of aspiration of the pharyngeal residues might occur. All patients were instructed to exhale before vacuum swallowing, and the most frequent patterns were E-SW and SW-E. These participants had a minimal risk concerning aspiration by respiration. In contrast, the I-SW pattern was observed in 38.9% of the cases of vacuum swallowing in healthy individuals using the SBMS. Even in some healthy individuals, a higher occurrence rate of I-SW/SW-I patterns was observed [27, 28]. However, during the instruction of vacuum swallowing, these I-SW/SW-I patterns would be better avoided to prevent aspiration of the pharyngeal residues. Holding one’s breath to close the airway before vacuum swallowing would be useful for preventing aspiration as supraglottic swallowing [29]. The common aspects of supraglottic and vacuum swallowing are those before and after swallowing, which is performed as an exhalation to prevent aspiration of hypopharyngeal residues in patients with dysphagia. Exhalation after vacuum swallowing would be particularly important. Vacuum swallowing differed from supraglottic swallowing since it combines inspiratory effort immediately after the swallow reflex occurs.

Furthermore, healthy individuals showed respiratory pause due to closed glottis during vacuum swallowing. Instruction of exhalation - holding breath – vacuum swallowing - expiration pattern would be important for airway protection from the aspiration risk of hypopharyngeal residues due to dysphagia, such as bulbar type. Forceful inspiration against glottic obstruction reportedly could result in a maximum intrathoracic pressure of -140 cmH_2_O (baseline, -4 cmH_2_O) [30]. Furthermore, an inspiratory effort with the closed glottis and pharyngeal constriction during swallowing would generate a strong subatmospheric negative pressure in the thoracic cavity. Therefore, the mechanism of vacuum swallowing involves the combination of swallowing movements and forceful inspiration.

Vacuum swallowing may be helpful as an alternative swallowing method to clear pharyngeal residues in the pyriform sinus. An indication for vacuum swallowing would be pharyngeal swallowing dysfunction with reduced pharyngeal contraction and impaired UES opening (e.g., bulbar-type dysphagia, including LMS) [15–19]. However, this study did not examine the impact of vacuum swallowing on the esophagus. Therefore, future studies should examine bolus flow in the esophagus using VF and HRM with impedance and determine whether the instruction of vacuum swallowing is effective for patients with specific types of dysphagia.

This study had some limitations. First, only healthy individuals were enrolled in this study. Therefore, further study should examine its effectiveness in patients with dysphagia. Second, HRM is only available in some facilities; therefore, establishing a method to confirm strong subatmospheric negative pressure in the esophagus is necessary. Third, the instructional method employed was slightly complicated. Therefore, preparing a simple and easy instruction manual and video for patients with dysphagia is necessary. Fourth, the sample size was small. Despite the limitations of this study, we determined that healthy individuals could reproduce vacuum swallowing. Fifth, this study could not confirm that penetration/aspiration was not associated with vacuum swallowing. Therefore, when instructing a patient with dysphagia to perform vacuum swallowing, the VF should be conducted to evaluate the penetration/aspiration. Once safety is confirmed, swallowing could be incorporated as one of the swallowing methods in clinical practice.

## Conclusions

Healthy individuals can generate a strong subatmospheric negative pressure in the esophagus during swallowing. However, further study is necessary to determine the dysphagic population that would benefit from this novel technique known as vacuum swallowing. Moreover, an easy-to-understand teaching method should also be evaluated in further studies with more individuals and patients with dysphagia.

## Electronic Supplementary Material

Below is the link to the electronic supplementary material.

Supplementary Material 1
